# Maternal Subclinical Hypothyroidism in Rats Impairs Spatial Learning and Memory in Offspring by Disrupting Balance of the TrkA/p75^NTR^ Signal Pathway

**DOI:** 10.1007/s12035-021-02403-z

**Published:** 2021-05-08

**Authors:** Fan Zhang, Xinyue Lin, Aihua Liu, Jian Chen, Zhongyan Shan, Weiping Teng, Xiaohui Yu

**Affiliations:** 1 Department of Endocrinology and Metabolism, Institute of Endocrinology, NHC Key Laboratory of Diagnosis and Treatment of Thyroid Diseases, The First Hospital of China Medical University, No. 155 Nanjing North Street, Shenyang, 110001 China; 2 Department of Endocrinology and Metabolism, Peking University Third Hospital, Beijing, 100191 China

**Keywords:** Subclinical hypothyroidism, Offspring, Hippocampus, Levothyroxine, TrkA/p75^NTR^ balance

## Abstract

Maternal subclinical hypothyroidism (SCH) during pregnancy can adversely affect the neurodevelopment of the offspring. The balance of nerve growth factor (NGF)-related tropomyosin receptor kinase A/p75 neurotrophin receptor (TrkA/p75^NTR^) signaling in the hippocampus is important in brain development, and whether it affects cognitive function in maternal SCH’s offspring is not clear. In this study, we found that compared with the control (CON) group, expression of proliferation-related proteins [NGF, p-TrkA, phospho-extracellular signal-regulated kinase 1/2 (p-ERK1/2) and phospho-cAMP response element-binding protein (p-CREB)] decreased in the hippocampus of the offspring in the SCH group, overt hypothyroidism (OHT) group, and the group with levothyroxine (L-T_4_) treatment for SCH from gestational day 17 (E17). In contrast, expression of apoptosis-related proteins [pro-NGF, p75^NTR^, phospho-C-Jun N-terminal kinase (p-JNK), p53, Bax and cleaved caspase-3] was increased. The two groups with treatment with L-T_4_ for SCH from E10 and E13, respectively, showed no significant difference compared with the CON group. L-T_4_ treatment enhanced relative expression of NGF by increasing NGF/proNGF ratio in offspring from maternal SCH rats. In conclusion, L-T_4_ treatment for SCH from early pregnancy dramatically ameliorated cognitive impairment via TrkA/p75^NTR^ signaling, which involved activation of the neuronal proliferation and inhibition of neuronal apoptosis in SCH rats’ offspring.

## Introduction

Thyroid hormones (THs) are essential for brain development and have a critical role in fetal brain development [1, 2]. TH deficiency during development can cause obvious and irreversible damage to neurodevelopment unless hormone replacement therapy is initiated on time [3]. Subclinical hypothyroidism (SCH) is the most common form of thyroid dysfunction in pregnancy with a prevalence of 3–5% [4, 5]. In the past few decades, epidemiological investigations have revealed that maternal SCH during pregnancy may have an impact on brain development in the offspring [6–8]. In the light of this phenomenon, our group found that the neurodevelopment of offspring that were born from rats with SCH during pregnancy was significantly worse than that from normal control rats, based on results from Morris water-maze (MWM) and long-term potentiation (LTP) [9–13]. We have also reported that the expression of brain-derived neurotrophic factor (BDNF) and its downstream molecules are altered in the offspring of maternal rats with SCH [11, 12]. Nerve growth factor (NGF) and BDNF are classical neurotrophic factors that regulate cell differentiation, proliferation, and survival; promote axon and dendritic growth; enhance synaptic plasticity; and inhibit apoptosis [14, 15]. NGF mainly binds to tropomyosin receptor kinase (Trk)A, which activates cAMP response element-binding protein (CREB) to regulate regeneration, survival, and proliferation of neurons [16]. Meanwhile, the p75 neurotrophin receptor (p75^NTR^) is a low-affinity receptor for NGF and promotes neuronal apoptosis by activating the C-Jun N-terminal kinase (JNK) signaling pathway [17, 18]. NGF plays a critical role in neurodevelopment by activating the TrkA signaling pathway and inhibiting the p75^NTR^ signaling pathway [14, 19–22]. Reduction of NGF in the cerebral cortex and cerebellum has been detected in the offspring of rats with perinatal hypothyroidism. This leads to inhibition of the TrkA signaling pathway, activation of the p75^NTR^ signaling pathway, and imbalance in the TrkA/p75^NTR^ signaling pathway [23, 24]. However, whether an imbalance of the TrkA/p75^NTR^ signaling pathway is found in hippocampal development in offspring of SCH rats has not been clarified.

The present study was designed to determine whether the TrkA/p75^NTR^ signaling pathway was imbalanced in the hippocampus of the offspring of maternal SCH rats, and to study the effects on the brain development of levothyroxine (L-T_4_) replacement therapy started at different times during pregnancy. Finally, we offer a theoretical basis for clinicians to choose the optimal intervention time for SCH women during pregnancy.

## Materials and methods

### Animals

Ninety nulliparous female Wistar rats (weighing 190–210 g) were housed in cages in a climate-controlled, specific-pathogen-free environment (23–25 °C, 55% relative humidity, 12-h light/dark photoperiod) with free access to normal rat chow and tap water in the Experimental Animal Department of China Medical University. All animals and experimental procedures were approved by the Animal Care and Use Committee of China Medical University, which complied with the National Institutes of Health Guide for the Care and Use of Laboratory Animals (NIH Publications No. 8023, revised 1978). After a 1-week adaptation period, we randomly divided all rats into two groups: 15 of 90 underwent sham thyroid surgery as the control (CON) group, and others underwent thyroidectomy, all operation were done after intraperitoneal (i.p.) injection of 3% pentobarbital sodium (0.1 mL/100 g). Calcium lactate (0.1% w/v) was added to the drinking water for all rats after surgery. One month later, 15 of 75 rats after thyroidectomy were randomly selected as the overt hypothyroidism (OHT) group. The remaining 60 rats were used to establish SCH models by subcutaneous (s.c.) injection of L-T4 (Sigma-Aldrich, St. Louis, MO, USA) in the neck at a concentration of 1.0 μg/100 g/day. Rats in the CON and OHT groups received s.c. injection of physiological saline (50 µL/100 g/day) in the same position. After 9 days, all 90 female rats copulated with normal male rats (male: female = 1: 2). The next morning, if sperm was found in vaginal smears, the day was set as day 0 of pregnancy (E0). The 60 thyroidectomized rats that received L-T_4_ injections were further randomly divided into four groups of 15: the SCH group continued L-T_4_ injections (1.0 μg/100 g/day), and other rats received an L-T_4_ replacement treatment (1.25 µg/100 g/day) to maintain normal thyroid hormone levels starting from E10, E13, and E17, respectively. The female rats were observed daily until they gave birth, and the delivery day was recorded as postnatal day 0 (PND0). All offspring weaned from PND21 and the female rats stopped subcutaneous injection meanwhile.

**Fig. 1:**
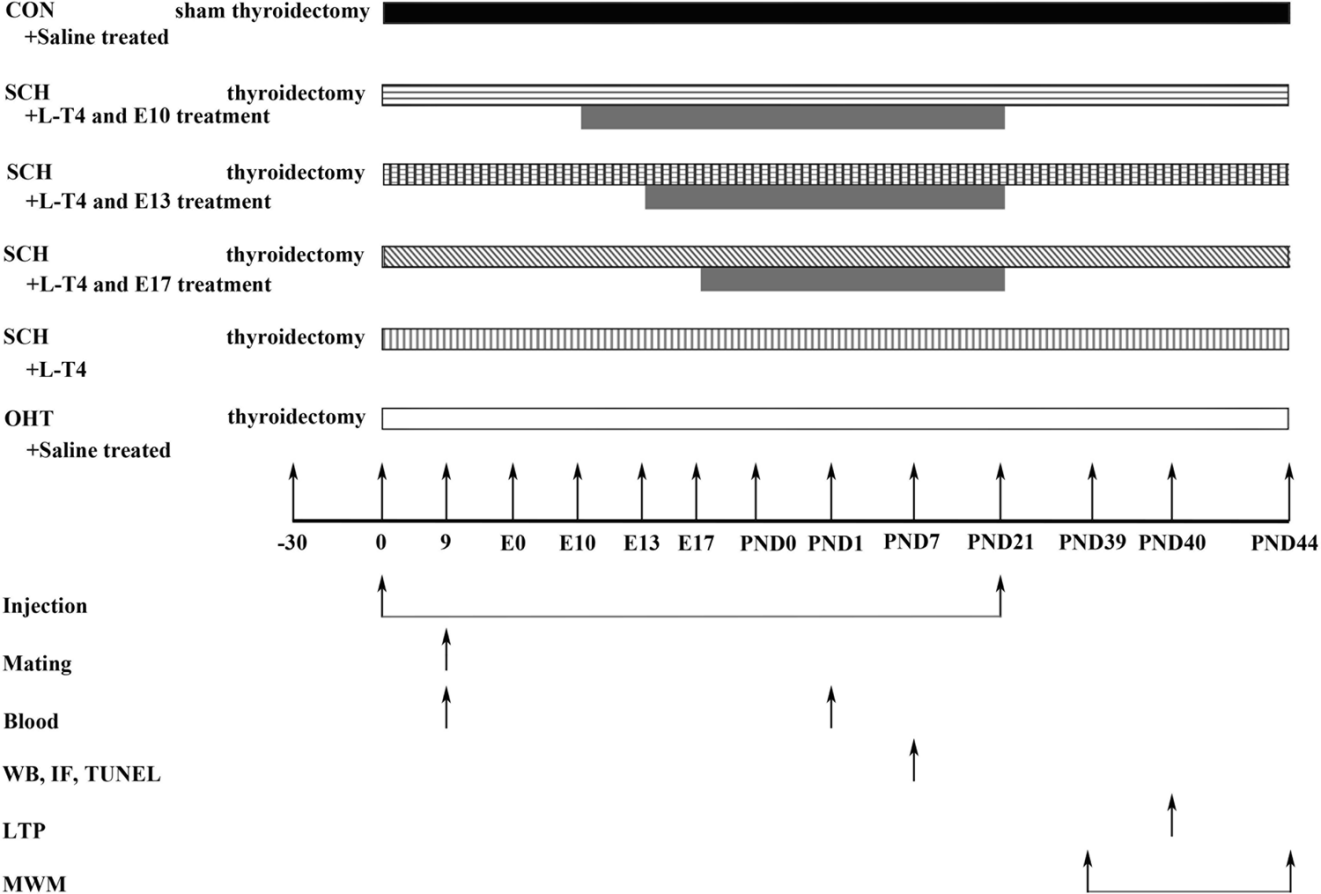
Schematic diagram of experimental timeline. E0, gestational day 0; IF, immunofluorescence; LTP, long-term potentiation; MWM, Morris water maze; PND, postnatal day; TUNEL, terminal deoxynucleotidyl transferase-mediated dUTP nick end-labeling; WB, western blotting

Blood samples (approximately 2 mL) were collected from the orbital vein of all pregnant rats 9 days after injection and PND0 to test the level of total thyroxine (TT_4_) and thyroid-stimulating hormone (TSH). On PND7, the pups were decapitated on ice for western blotting (N = 6), immunofluorescence and terminal deoxynucleotidyl transferase-mediated dUTP nick end-labeling (TUNEL) staining (N = 6). The remaining pups were raised to PND39 for MWM tests (*n* = 10) and LTP induction assay (*n* = 6). The project timeline is described in Fig. 1.

### Measurements of TT_4_ and TSH

Blood samples obtained from rats were centrifuged at 20,913 g for 15 min immediately and stored at − 80 °C for subsequent measurement including TT_4_ and TSH. TT_4_ was calculated by Roche ElectroChemiLuminescence (Roche Diagnostic Products, Los Angeles, CA, USA). TSH was measured by ELISA Kit for Thyroid Stimulating Hormone (Cloud-Clone Corp., Houston, TX, USA). The inter- and intra-assay coefficients of variation (CVs) for TT_4_ were 3.3–4.2 and 1.3–1.8%, respectively. The inter- and intra-assay CVs for TSH were < 10 and < 12%, respectively. All samples for TT_4_ and TSH measurements were performed in duplicate.

### MWM test

The MWM test (including navigation tests and a probe trial test) was utilized to appraise the degree of spatial learning in pups from all groups. The MWM consisted of a black circular swimming pool (100 cm diameter × 60 cm depth) divided into four equal quadrants (I–IV) by two straight lines. A circular platform (10 cm diameter) was placed in the middle of quadrant IV and was sunk 1 cm below the surface of the water. Starting from PND39, the pups were randomly selected from each group to finalize the test (N = 10). On the first day, the pups were allowed to swim freely for 120 s to adapt to the water maze and platform. On the second day (PND40), the pups started training; each pup was trained four times from the four different quadrants per day. The time of pups finding the platform was defined as escape latency. Once the pup climbed on the platform, escape latency was recorded and the pup was recycled gently after staying on the platform for 10 s. If a pup failed to find the platform after 120 s, it was guided to the platform and the escape latency was registered as 120 s. We repeated the aforementioned training until the fifth day (PND40, PND41, PND42, and PND43). On the sixth day, the probe trial was performed to assess long-term memory. The platform was removed and the experiments performed on the previous 4 days were repeated. We recorded the number of times each pup reached the platform area and the time spent in the area, which was assigned as the probe trial.

### LTP induction

Synaptic plasticity in the CA1 area of the hippocampus at 6 weeks can be evaluated by high-frequency stimulation (HFS)-induced increase in field-excitatory postsynaptic potentials (f-EPSPs). We measured f-EPSPs in the hippocampus of the pups from each group using a MED64 planar microelectrode matrix recording system (Alpha Med Science, Osaka, Japan). Pups were anesthetized with 3% sodium pentobarbital (30 mg/kg, i.p.) on PND40 (N = 6). The brains were immediately removed and cut into 300-μm-thick slices. Brain slices were incubated in artificial cerebrospinal fluid (ACSF): 14.5 g NaCl, 0.44 g KCl, 4.36 g NaHCO_3_, 0.24 g MgSO_4_, 0.34 g KH_2_PO_4_, 3.6 g D-glucose, and 0.44 g CaCl_2_ in 2 L deionized water (pH 7.35–7.45). The temperature of ACSF was maintained at 33–35 °C and the flow rate at 1–1.5 mL/min. Simultaneously, the mixed gas containing 95% O_2_ and 5% CO_2_ passed to maintain these brain slices. The baseline level before HFS was measured and stabilized for 10–15 min. Input/output curves were obtained by increasing the intensity of the stimulus and adjusting it to elicit 70% of the maximum response. The f-EPSP was recorded and the stimulus value corresponding to a 50% amplitude difference was recorded as the HFS. The tissue was stimulated twice with HFS, and LTP was induced after 10 s and recorded for > 30 min. The f-EPSP% increase after HFS was used as an indicator for evaluating LTP.

### Western blotting

On PND7, the pups were sacrificed on ice after anesthesia (3% sodium pentobarbital, 0.1 mL/100 g, i.p.) (N = 6). The hippocampus was removed immediately and every 0.1 g hippocampus tissue was added to 500 μL buffer containing protease phosphatase inhibitors and phenylmethanesulfonylfluoride (Keygen Biotech, Nanjing, China). The tissue was homogenized by shaking and centrifugation at 20,931 g for 15 min at 4 °C. Protein concentrations were determined by the bicinchoninic acid method (Beyotime, Shanghai, China), and the samples were stored at − 80 °C. Tissue lysates were diluted to the same protein concentrations (3 μg/μL), boiled for 8 min, and 10-μL (30 g protein) samples from each group were subjected to 10% SDS-PAGE. The markers were separated at a constant 80 V for 30 min, and the proteins were separated at a constant 120 V for 1 h. After electrophoresis, the proteins were transferred to polyvinylidene difluoride membranes (Millipore, Billerica, MA, USA) at a constant of 100 V for 1.5 h. Nonspecific binding was blocked using a mixture of Tris-buffered saline and 0.05% Tween-20 with the skimmed milk powder, except for phospho-C-Jun N-terminal kinase (p-JNK), phospho-cAMP response element-binding protein (p-CREB), phospho-extracellular signal-regulated kinase (p-ERK) and phospho-tropomyosin receptor kinase A (p-TrkA) that were blocked with bovine serum albumin (BSA). The membranes were incubated overnight at 4 °C with the following antibodies: rabbit anti-NGF (1:1000 dilution; Abcam, ab52918, Cambridgeshire, UK); rabbit anti-proNGF (1:1000 dilution; Sigma-Aldrich, P5498); rabbit anti-total TrkA (1:1000 dilution; Cell Signaling Technology 2505S, USA); rabbit anti-p-TrkA (1:1000 dilution; Abcam, ab1445); rabbit anti-total ERK (1:1000 dilution; CST4695S); rabbit anti-p-ERK (1:2000 dilution; CST4370S); rabbit anti-total CREB (1:1000 dilution; CST9197S); rabbit anti-p-CREB (1:1000 dilution; CST9198S); rabbit anti-p75^NTR^ (1:1000 dilution; CST8238S); rabbit anti-p53 (1:1000 dilution; CST32532S); rabbit anti-total JNK (1:1000 dilution; CST9252S); rabbit anti-p-JNK (1:1000 dilution; CST4668S); rabbit anti-Bax (1:1000 dilution; CST2772S); and rabbit anti-cleaved caspase-3 (1:1000 dilution; CST9664S). The blots were incubated for 1.5 h with horseradish-peroxidase-conjugated secondary antibody (1:5000 dilution; ZB2301, Beijing, China) and developed by chemiluminescent western blotting (ALPHAVIEW, version 1.3; Protein Simple Inc., San Jose, CA, USA). The optimal time to expose the blot to the membrane was determined by standardization experiments.

### Immunofluorescence

On the PND7, pups were transcardially perfused with saline and then with 4% paraformaldehyde solution at pH 7.4 (N = 6). The brains were removed from the skull, fixed with the same fixative for 5 days, and transferred to 10, 20, and 30% sucrose solution for dehydration for 2 days each. After dehydration was completed, the brains were cut into 10-μm-thick pieces using a freezing microtome (− 20 °C, CM1950; Leica, Wetzlar, Germany) on glass slides and stored at − 20 °C. Four random sections were obtained from each pup’s brain. The tissue sections were washed three times in phosphate-buffered saline with 0.05% Tween-20 (PBST) and incubated in a blocking solution containing 5% BSA and 0.3% Triton X-100 at room temperature for 1 h. These tissue sections were incubated overnight with primary antibodies: rabbit anti-p-CREB (1:400 dilution; CST9198S), rabbit anti-cleaved caspase-3 (1:800 dilution; CST9664S) and mouse anti-NeuN (1:500 dilution; NBP1-92,693, Colorado, USA) at 4 °C. On the next day, after PBST washing, the sections were incubated for 2 h with fluorochrome-tagged specific secondary antibodies: Alexa Flour 488-conjugated Affinipure Goat Anti-Rabbit IgG(H + L) (1:300 dilution; SA00013-2, Chicago, Proteintech); CoraLite 594-conjugated Goat Anti-Rabbit IgG(H + L) (1:100 dilution; CL594-66,467, Proteintech); and AMCA-conjugated Affinipure goat anti-mouse IgG(H + L) (1:20 dilution; SA00010-1, Proteintech) at room temperature away from light. Finally, the sections were mounted in an anti-fade mounting medium and examined hippocampus CA1 by confocal microscopy (20X, Leica TCS SP5 II, Germany).

### TUNEL staining

On the PND7, TUNEL staining was performed on the frozen tissue sections using the In Situ Cell Death Detection Kit, POD (11,684,817,910, Roche, Sigma, USA). The sections were fixed in freshly prepared 4% paraformaldehyde solution at pH 7.4 for 1 h at 15–25 °C. The sections were blocked in 3% H_2_O_2_ for 10 min at 15–25 °C, after rinsing three times with PBS. The sections were permeabilized by freshly prepared 0.1% Triton X-100 in 0.1% sodium citrate for 2 min on ice (2–8 °C), after rinsing three times with PBS. Sections were rinsed twice with PBS and we dried the area around the sample, followed by incubation with TUNEL reaction mixture for 1 h at 37 °C in a humidified atmosphere in the dark. The sections were rinsed three times with PBS and analyzed in a drop of PBS under confocal microscopy to exam hippocampus CA1 (20X, Leica TCS SP5 II), after being counterstained with 1 µg/mL 4′,6-diamidino-2-phenylindole (DAPI) dye.

### Statistical analysis

Data processing and statistics were conducted using SPSS 22.0 software (SPSS, Chicago, IL, USA). The results were expressed as the mean ± standard deviation (SD). Multiple group comparisons were performed using ANOVA followed by Dunnett’s T3 test. *p* < 0.05 was considered to be statistically significant. All artwork was created by GraphPad Prism 7.0 (La Jolla, CA, USA).

## Results

### TH levels in pregnant rats

**Fig. 2:**
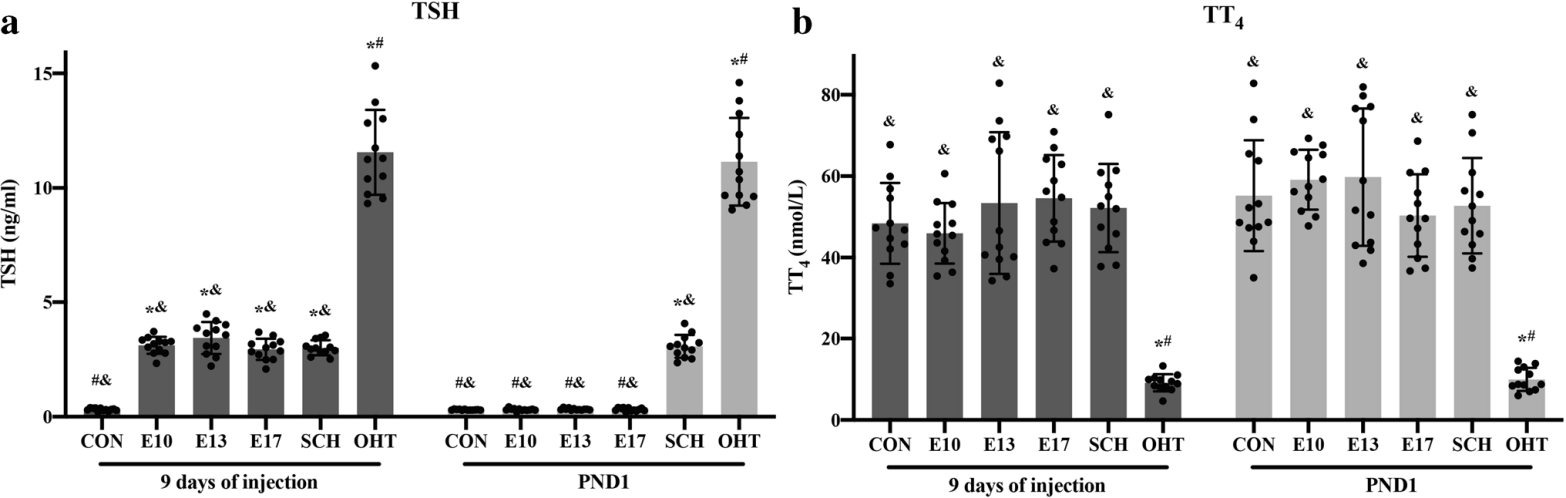
Thyroid-stimulating hormone (TSH) (**a**), and serum total thyroxine (TT_4_) (**b**) levels in pregnant rats (9 days of injection, N = 12; PND1, N = 12). ^*^p < 0.05 versus same day CON group; ^#^*p* < 0.05 versus same day SCH group; ^&^*p* < 0.05 versus same day OHT group. CON, control; PND1, postnatal day 1; OHT, overt hypothyroidism; SCH, subclinical hypothyroidism; TSH, thyrotropin; TT_4_, total thyroxine

We assessed serum TT_4_ and TSH levels in maternal rats at 9 days after L-T4 injection and PND1 to confirm the established success of the SCH, OHT, and L-T4 treatment models. The results of TSH and TT_4_ of each group of rats at 9 days of injection and PND1 were shown in Table 1. The OHT group showed a significant increase in TSH and decrease in TT_4_ compared with the CON group at 9 days after L-T4 injection and PND1. Compared with the CON group, TSH levels were significantly higher in SCH rats. Nevertheless, there was no significant difference in TT_4_ levels at 9 days after L-T4 injection and PND1. SCH pregnant rats’ serum TT_4_ levels showed significantly higher than the OHT group. Before L-T4 treatment, rats in the three treatment groups had a similar thyroid status compared with the SCH group. After L-T4 treatment at 1.25 μg/100 g/day, TSH levels of maternal rats in three treatment groups were suppressed significantly (*p* < 0.05) compared with the SCH group. No significant difference was observed in serum TT_4_ level between the CON and L-T4 treatment groups at 9 days after L-T4 injection and PND1. These results indicated that treatment with L-T4 was successful and additional L-T4 supplements recovered normal thyroid status in maternal rats. The SCH, OHT, and L-T4 treatment group models were established successfully throughout the pregnancy (Fig. 2).

**Table 1:** The thyroid-stimulating hormone (TSH) and serum total thyroxine (TT_4_) of each group maternal rats at 9 days of injection and PND1

Group	9 days of injection (N = 12)		PND1 (N = 12)	
	TSH (ng/mL)	TT_4_ (nmol/L)	TSH (ng/mL)	TT_4_ (nmol/L)
CON	0.32 ± 0.06 ^#&^	48.41 ± 9.94 ^&^	0.31 ± 0.02 ^#&^	55.23 ± 13.63 ^&^
E10	3.12 ± 0.37 *^&^	45.96 ± 7.43 ^&^	0.31 ± 0.05 ^#&^	59.13 ± 7.35 ^&^
E13	3.44 ± 0.70 *^&^	53.40 ± 17.43 ^&^	0.33 ± 0.04 ^#&^	59.76 ± 16.85 ^&^
E17	2.94 ± 0.46 *^&^	54.56 ± 10.62 ^&^	0.32 ± 0.07 ^#&^	50.31 ± 10.13 ^&^
SCH	3.01 ± 0.33 *^&^	52.19 ± 10.84 ^&^	3.07 ± 0.50 *^&^	52.74 ± 11.70 ^&^
OHT	11.56 ± 1.85 *^#^	9.14 ± 2.12 *^#^	11.15 ± 1.91 *^#^	9.97 ± 2.85 *^#^

^*^p < 0.05 versus same day CON group; ^#^*p* < 0.05 versus same day SCH group; ^&^*p* < 0.05 versus same day OHT group. *CON*, control; *PND1*, postnatal day 1; *OHT*, overt hypothyroidism; *SCH*, subclinical hypothyroidism; *TSH*, thyrotropin; *TT*_4_, total thyroxine

### The adverse effect of maternal SCH on spatial learning and memory in offspring

**Fig. 3:**
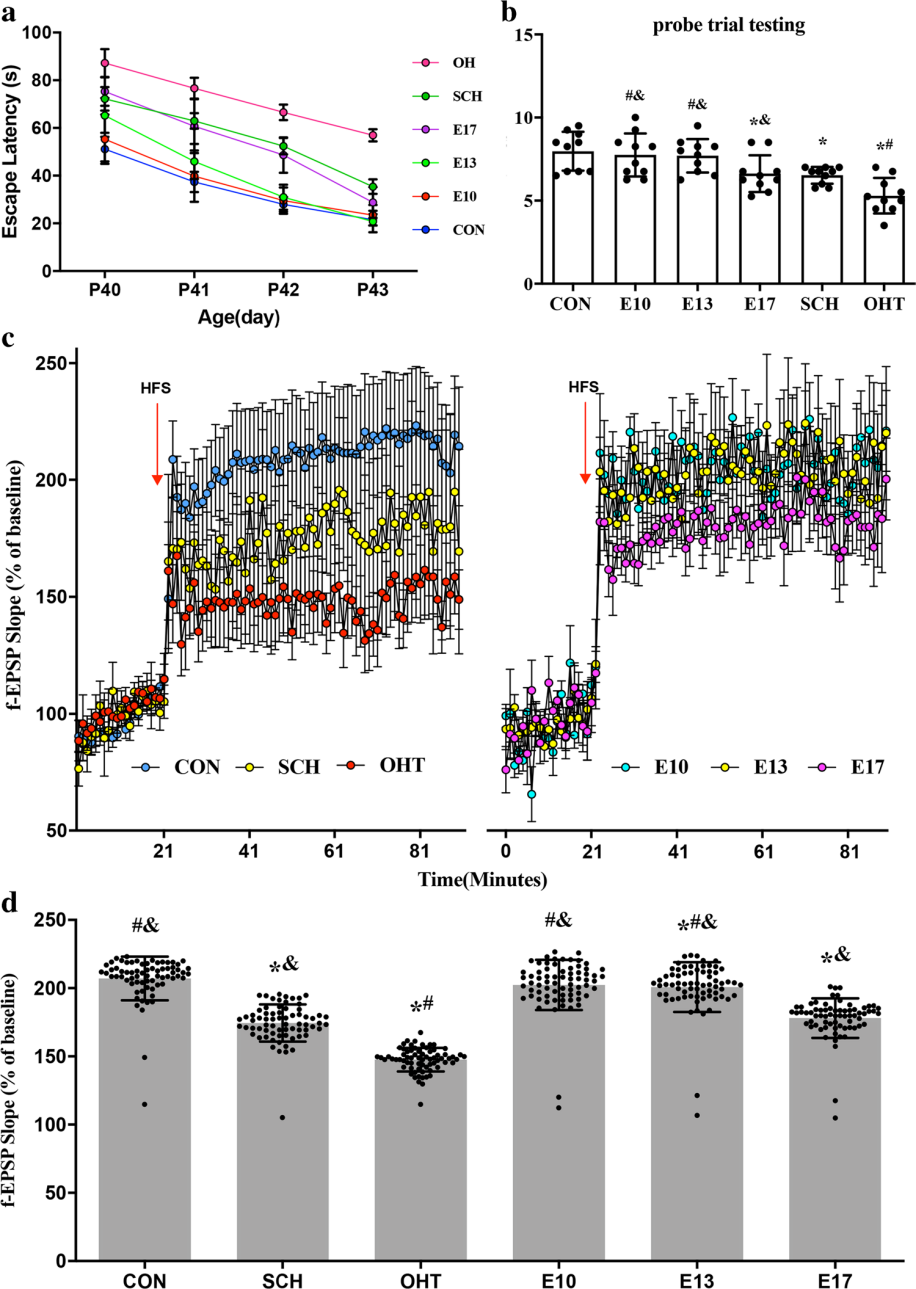
Performance of pups in the MWM test (N = 10, PND39-43) and LTP (N = 6, PND40). Data are expressed as the mean ± SD. **a**. Average time to find the hidden platform was longer in the SCH and OHT groups compared with the CON group from PND 40 to 43. **b**. Probe trial test recorded the number of times pups crossed the platform quadrant. **c**. Maternal SCH caused LTP damage in the hippocampal CA1 region in their pups. LTP was induced by HFS and measured as an increase in f-EPSP slope, expressed as a percentage of the baseline of the f-EPSP slope after HFS in all groups. f-EPSP slopes were reduced in the SCH and OHT groups compared with the CON group (*p* < 0.05). **d**. Intuitively represent the f-EPSP slope after HFS (% of baseline). ^*^p < 0.05 versus same day CON group; ^#^*p* < 0.05 versus same day SCH group; ^&^*p* < 0.05 versus same day OHT group. CON, control; E, gestational day; f-EPSP, field excitatory postsynaptic potential; HFS, high-frequency stimulation; LTP, long-term potentiation; MWM, Morris water maze; OHT, overt hypothyroidism; PND, postnatal day; SCH, subclinical hypothyroidism

MWM was used to evaluate spatial learning and memory. In all the offspring, escape latency decreased with increased training. Escape latency from PND40 to PND43 was significantly longer in the SCH group compared with the CON group, and that in the OHT group was the longest (*p* < 0.05). Compared with the SCH group, the latency of the three L-T_4_ treatment groups was shortened. Although there were significant differences between the E10 and E13 L-T_4_ treatment groups and the CON group on PND40 (*p* < 0.05), no significant differences on PND 41–43 (*p* > 0.05), and no significant difference between the E17 L-T_4_ treatment and SCH groups (*p* > 0.05). Starting from training, there was no significant difference in the escape latencies between the CON group and the E10 and E13 L-T4 treatment groups (*p* > 0.05). On the last day of training, there was no significant difference between the three L-T_4_ treatment groups and the CON group (Fig. 3a).

There was no significant difference in the number of times that the pups crossed the platform quadrant in the probe trial on PND44 between the SCH and E17 L-T_4_ treatment groups (*p* > 0.05). However, they were markedly lower than in the CON, E10, and E13 groups, but significantly higher than in the OHT group (*p* < 0.05) (Fig. 3b). These results showed that spatial learning and memory were impaired in the offspring from SCH rats. L-T_4_ treatment starting from E10 and E13 ameliorated the adverse effect of maternal SCH on spatial learning and memory in offspring.

### Decrease of LTP in offspring from maternal SCH rats

Spatial learning and memory require synaptic transmission, which is characterized as the LTP of f-EPSPs. The LTP results were evaluated by measuring baseline f-EPSP% after HFS. There were no significant differences in f-EPSP among all groups before HFS (*p* > 0.05), and all six groups showed a significant increase after HFS. The amplification percentage of the f-EPSP slope in the SCH and OHT groups was significantly lower than that in the CON group (*p* < 0.05). L-T_4_ treatment ameliorated the amplification percentage of the f-EPSP slope in the E10 and E13 groups compared with the CON group, and it was significantly higher than that of the SCH and OHT groups (*p* < 0.05). L-T_4_ treatment starting from E17 also increased the amplification percentage of the slope of the f-EPSPs compared with the percentage in the SCH group, but the difference was not significant. The slope of the f-EPSPs was still lower than that of the CON group (*p* < 0.05). Pups in the OHT group demonstrated the lowest levels of amplification percentage of the slope of the f-EPSPs (Fig. 3c–d). These results suggested that SCH during pregnancy damaged LTP in the offspring. L-T_4_ treatment on E10 and E13 could have prevented LTP decrease in SCH rat offspring.

### Expression of NGF, proNGF and calculated the NGF/proNGF ratio in offspring from SCH and L-T_4_ treatment groups

**Fig. 4:**
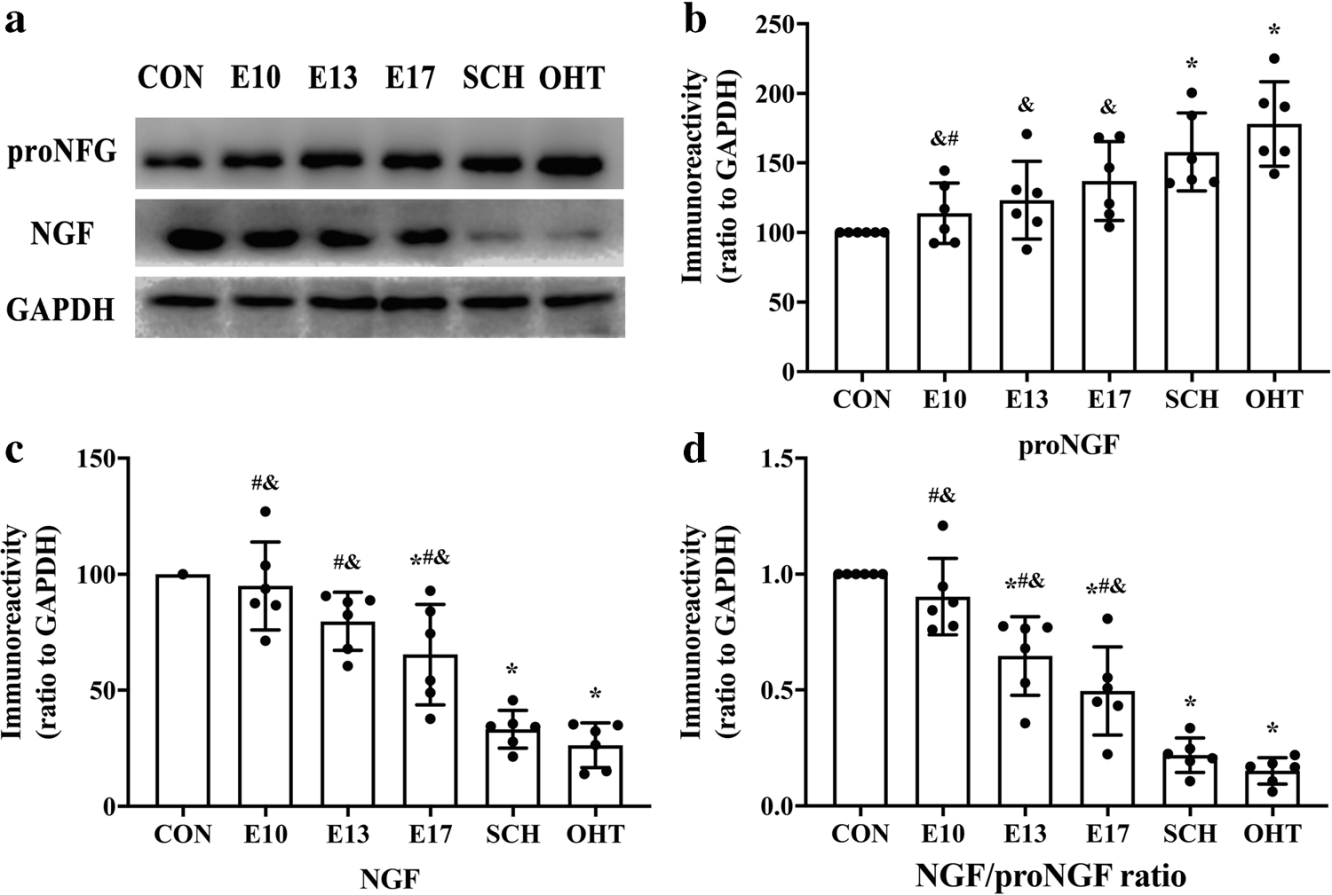
Expression of NGF (**a**, **c**) and its precursor neurotrophin pro-NGF (**a**, **b**), as well as the ratio of NGF and pro-NGF (**d**) in the hippocampus of pups (N = 6, PND7). ^*^p < 0.05 versus CON group, ^#^*p* < 0.05 versus SCH group, ^&^*p* < 0.05 versus OHT group. CON, control; E, gestational day; NGF, nerve growth factor; OHT, overt hypothyroidism; SCH, subclinical hypothyroidism

We measured the expression of NGF and proNGF and NGF/proNGF ratio in the hippocampus from all six groups. Western blotting showed that expression of proNGF increased in SCH (1.58-fold) and OHT (1.78-fold) groups’ offspring compared with the CON group (both *p* < 0.05). Compared with the SCH group, expression of proNGF was restored in the offspring of the E10 groups (*p* < 0.05). And L-T_4_ treatment in the three groups restored proNGF to levels that were not significantly different from those in the CON group (all *p* > 0.05). In the E17, SCH and OHT groups, NGF levels were significantly reduced to 65, 33 and 26% of the control levels, respectively (all *p* < 0.05). Compared with the SCH group, expression of NGF was restored to some extent in the rats treated with L-T4 from E10, E13, and E17 (all *p* < 0.05; Fig. 4a–c). NGF/proNGF ratio in the SCH and OHT groups were significantly lower than in the CON group (both *p* < 0.05; Fig. 4c), and the OHT group had the largest decline. In the E10 group, NGF/proNGF ratio returned to a level that was not significantly different from that in the CON group (*p* > 0.05; Fig. 4c). NGF/proNGF ratio in the E13 and E17 groups was still significantly lower than that in the CON group (*p* < 0.05; Fig. 4c). These results suggested that SCH during pregnancy could have unbalanced TrkA/p75^NTR^ in the hippocampus of offspring. Timely L-T_4_ treatment during pregnancy ameliorated the balance of TrkA/p75^NTR^ by augmenting the NGF/proNGF ratio.

### Expression of proliferation-associated NGF/TrkA pathway in offspring from SCH and L-T_4_ treatment groups

**Fig. 5:**
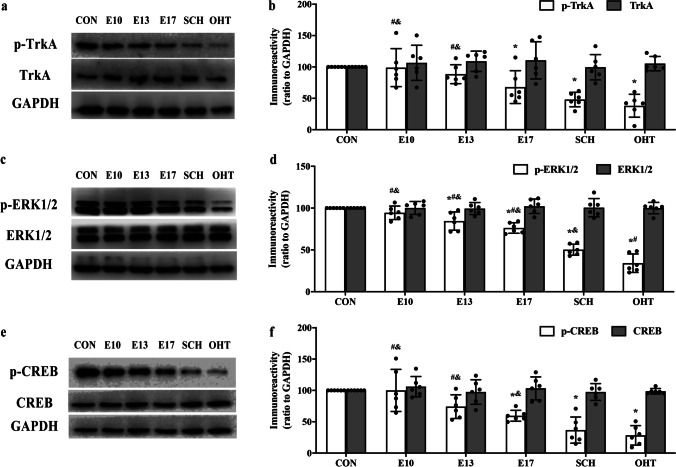
Expression of phospho-tropomyosin-related kinase A (p-TrkA) (**a, b**), phospho-extracellular signal-regulated kinase 1/2 (p-ERK1/2) (**c**, **d**) and phospho-cAMP response element-binding protein (p-CREB) (**e**, **f**) in the hippocampus of pups (N = 6, PND7). ^*^p < 0.05 versus CON group, ^#^*p* < 0.05 versus SCH group, ^&^*p* < 0.05 versus OHT group. CON, control; E, gestational day; OHT, overt hypothyroidism; SCH, subclinical hypothyroidism

We investigated the NGF/TrkA-related signaling pathway and related substrates downstream in offspring of SCH and L-T_4_ treatment groups. Western blotting showed that compared with the CON group, expression of p-TrkA (Fig. 5a, b), p-ERK1/2 (Fig. 5c, d), and p-CREB (Fig. 5e, f) were decreased in the hippocampus of offspring from SCH and OHT rats (all *p* < 0.05), but the total protein levels had no significant differences, respectively (all *p* > 0.05, Fig. 5c, f, i). Compared with the SCH group, the expression of p-TrkA and p-CREB increased significantly in L-T_4_ treatment from E10 and E13 (*p* < 0.05), and three L-T_4_ treatment groups p-ERK1/2 were increased significantly (*p* < 0.05). Nevertheless, the total protein levels had no significant differences among all six groups (*p* > 0.05).

Immunofluorescence showed that expression of p-CREB was consistent with that in western blotting (Fig. 7a, d). These results indicated that SCH during pregnancy reduced expression of proliferation-associated NGF/TrkA pathway in the hippocampus of offspring and decreased neuronal proliferation. Treatment with L-T_4_ treatment from E10 and E13 reversed the decrease in NGF/TrkA pathway in offspring of SCH rats.

### Expression of apoptosis-associated proNGF/p75^NTR^ pathway in offspring from SCH and L-T4 treatment groups

**Fig. 6:**
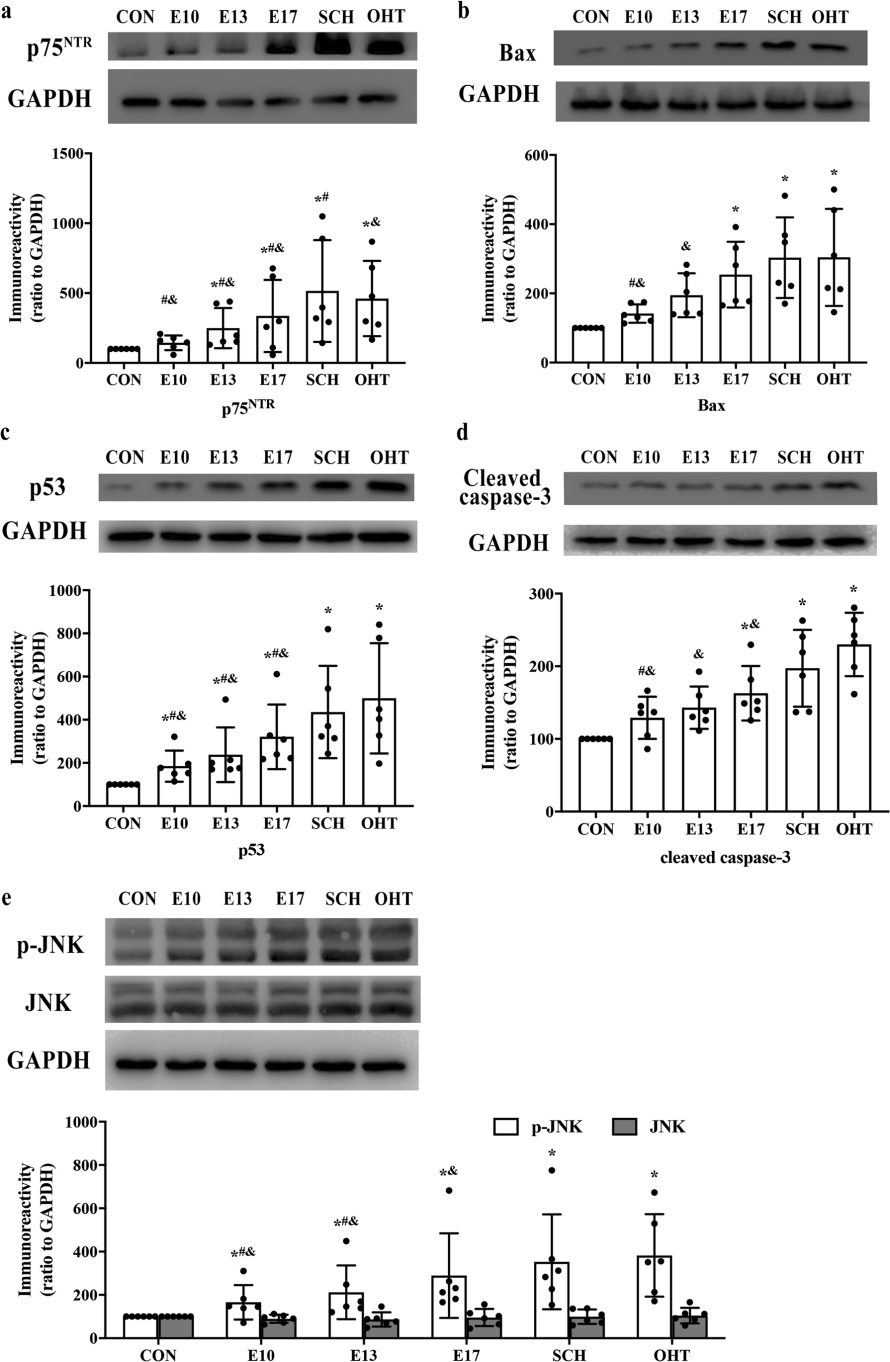
Expression of p75 neurotrophin receptor (p75^NTR^) (**a**), Bax (**b**), proapoptotic proteins p53 (**c**) and cleaved caspase-3 (**d**), and phospho-c-Jun N-terminal kinase (p-JNK) (**e**) in the hippocampus of pups (N = 6, PND7). ^*^p < 0.05 versus CON group, ^#^*p* < 0.05 versus SCH group; ^&^*p* < 0.05 versus OHT group. CON, control; E, gestational day; OHT, overt hypothyroidism; SCH, subclinical hypothyroidism

We investigated the proNGF/p75^NTR^-related signaling pathway and related substrates downstream in offspring of SCH and L-T_4_ treatment groups. Western blotting showed that, compared with the CON group, p75^NTR^ (Fig. 6a), Bax (Fig. 6b), p53 (Fig. 6c), cleaved caspase-3 (Fig. 6d), and p-JNK (Fig. 6e) were overexpressed in pups from the SCH and OHT groups. In the hippocampus of offspring from the three L-T_4_ treatment groups (E10, E13, and E17), protein expression was increased to varying degrees. However, the total JNK levels were similar among all groups (Fig. 6i and k).

**Fig. 7:**
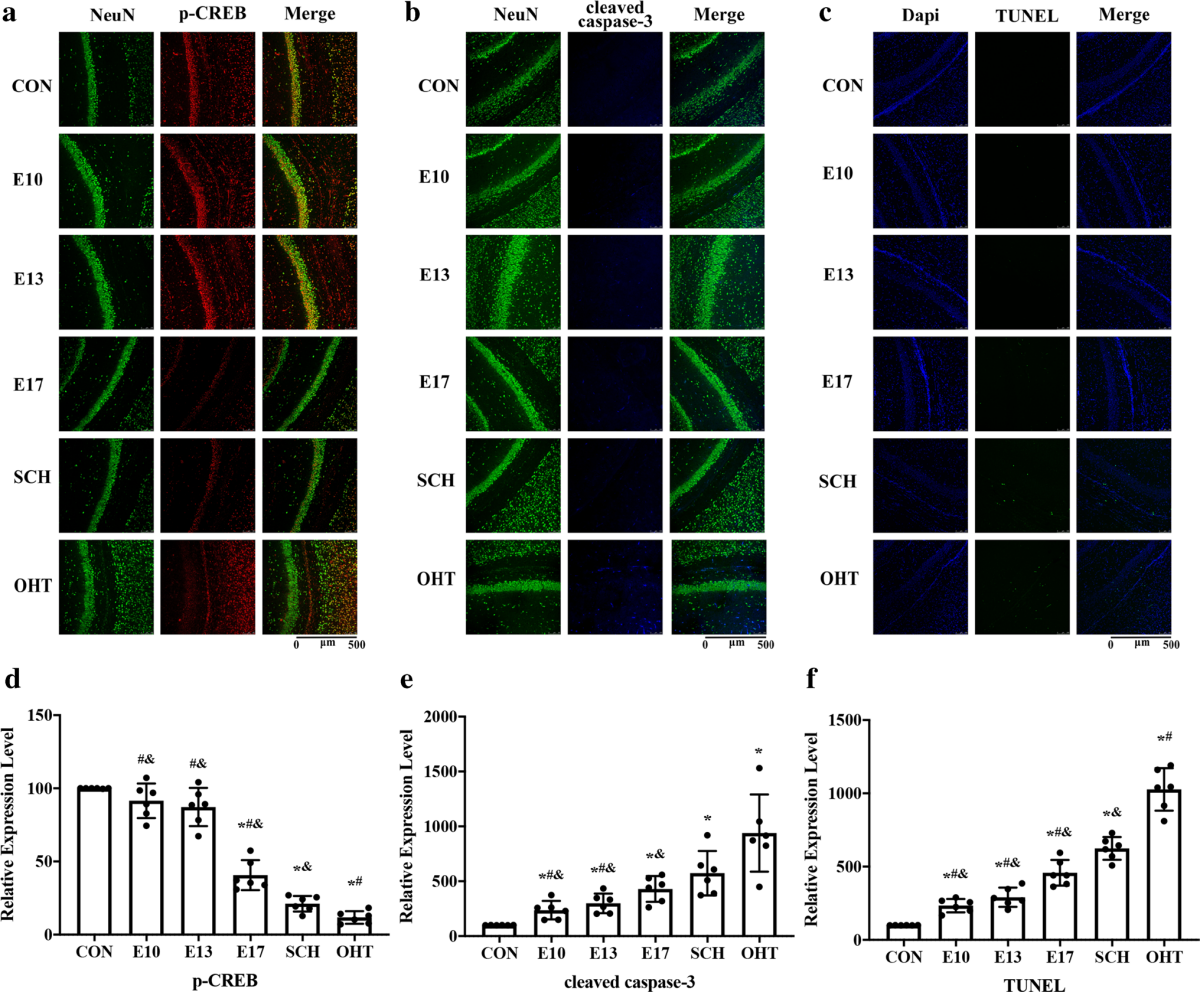
The results of immunofluorescence and TUNEL staining in the hippocampus of pups (N = 6, PND7). **a**. Double immunofluorescence with antibodies against NeuN (green) and phospho-cAMP response element-binding protein (p-CREB) (red) demonstrated the expression of p-CREB on hippocampal neurons. **b**. Double immunofluorescence with antibodies against NeuN (green) and cleaved caspase-3 (blue) demonstrated the expression of cleaved caspase-3 on hippocampal neurons. **c**. The neural apoptosis in the hippocampus, as shown by TUNEL assay (TUNEL, green; DAPI, blue). The merged images showed overlapping localization. **d**–**f**. Quantitative analysis of the relative expression level expression of p-CREB, cleaved caspase-3 and TUNEL^**+**^. Scale bar = 500 μm. TUNEL, terminal deoxynucleotidyl transferase-mediated dUTP nick end-labeling

The SCH group showed less expression of cleaved caspase-3 than the OHT group but higher expression than the CON and L-T4 treatment groups (Fig. 7b, e). Consistent with the cleaved caspase-3 results, the number of TUNEL-positive cells in the hippocampus was significantly increased in the SCH and OHT groups compared with the CON group. Offspring in the L-T4 treatment groups showed fewer TUNEL-positive cells in the hippocampus than those in the SCH and OHT groups (Fig. 7c, f). These results indicated that SCH during pregnancy activated expression of the apoptosis-associated NGF/75^NTR^ pathway in offspring to increase neuronal apoptosis. L-T4 treatment on E10 and E13 reversed the increase in the NGF/p75^NTR^ pathway in offspring of SCH rats.

## Discussion

Previous studies have demonstrated that maternal SCH can adversely affect the neurodevelopment of offspring, both in humans [6, 8] and animals [9–13]. Our group has found that many neurodevelopment-related proteins in the progeny of maternal SCH rats are reduced, such as BDNF, p-ERK, p-CREB, p-CaMKIV, p75^NTR^ [11–13]. This study continued to explore the mechanism of impaired neurocognitive ability caused by maternal SCH that imbalance of NGF-related TrkA/p75^NTR^ signaling in the hippocampus of offspring in SCH rats.

There are two main stages in the prenatal development of mammals in terms of TH synthesis and release into the fetal nervous system. In stage I, the first trimester of pregnancy, fetal brain development requires a large number of THs from the maternal coelomic and/or amniotic fluid [25]. In stage II, the fetal thyroid begins to develop, and the THs from the fetus and mother work together [25, 26]. At birth, the brain development of the fetus is dependent entirely on the THs secreted by the neonatal thyroid [27]. We selected gestational time points in rats that corresponded to those in humans. The fetal thyroid does not begin to develop until E11 and serves a useful function by E17 [28]. Therefore, we chose E10 of rats as equivalent to week 8 of a human pregnancy, E13 as week 12 and E17 as week 16. We used thyroidectomy combined with supplementation with L-T_4_ to establish the SCH and L-T_4_ treatment group models. The dose of L-T4 was based on the results of serum thyroid function indexes, which is consistent with previous studies [11].

Compared with the CON group, spatial learning and memory in the SCH group pups were significantly decreased when evaluated by the MWM test. These impairments were more severe in the OHT group. Additionally, L-T_4_ treatment from E10 and E13 improved the impairments close to the levels of the CON group. These results are in agreement with the study of Wang et al. [11]. Our results also showed that more training sessions had a greater effect on escape latencies. The probe trial test showed that, compared with the CON group, SCH and OHT groups’ pups had impairment of sustained memory, which was restored by L-T_4_ treatment from E10 and E13. These results suggested that the progeny of maternal SCH and OHT rats may have irreversible damage to neurodevelopment, and the neural network connectivity cannot be restored even after prolonged training.

Another method to assess the ability of learning and memory is measuring synaptic plasticity by LTP in the hippocampus of pups. The amplitude and f-EPSP slope in the CON group were significantly higher than those in the SCH and OHT groups, which indicated damaged LTP. LTP in the E10 and E13 L-T_4_ treatment groups showed that f-EPSP increased significantly compared with the SCH group. This demonstrated that L-T_4_ treatment from E13 at least could moderate the effect of SCH on offspring’s synaptic plasticity, which was in line with previous studies [11].

TH deficiency can result in the reduction of the number of neurons, affect the course of neuronal proliferation and migration, reduce synaptic plasticity, and increase neuronal apoptosis in rodents. These factors may be the potential mechanism by which SCH impairs spatial learning and memory [13, 29, 30]. These physiological processes are further regulated by NGF [31–34]. It is known that NGF can activate the NGF/TrkA signaling pathway and function in neuronal proliferation and migration as well as synaptic plasticity. The NGF/p75^NTR^ signaling pathway can be suppressed by TrkA activation to elicit antiapoptotic effects. In contrast, as the precursor protein of NGF, proNGF can bind to p75^NTR^ easier than NGF and elicit neuronal apoptosis [35–38]. ProNGF can switch between neurotrophic and apoptotic activity by responding to changes in TrkA receptor levels, whereas mature NGF cannot [39]. Casponi et al. found that, as NGF was neutralized by the recombinant antibody in AD11 anti-NGF transgenic mice, proNGF was predominant, which led to p75^NTR^-dependent apoptosis and cognitive deficits. The p75^NTR^-dependent apoptosis and cognitive defect could be fully reversed by NGF supplementation [37]. Therefore, the relative expression level of NGF and proNGF (NGF/proNGF ratio) plays an important role in maintaining the TrkA/p75^NTR^ balance [40]. Once TrkA/p75^NTR^ are out of balance, apoptosis is higher than the proliferation of neurons, which is not conducive to the development of the nervous system, and results in the suboptimal neurocognitive ability of the offspring. Our results showed that due to the lack of NGF, the ratio of NGF/proNGF was imbalanced, which prompted an imbalance of the TrkA/p75^NTR^ pathways. In turn, it disrupted the balance of neuronal proliferation and apoptosis, and impaired the development of memory and learning ability of the offspring from maternal SCH. Moreover, our results showed that timely supplementation with L-T_4_ in SCH rats can activate the neuroprotective effects by adjusting the TrkA/p75^NTR^ imbalance status in pups’ neurodevelopment.

**Fig. 8:**
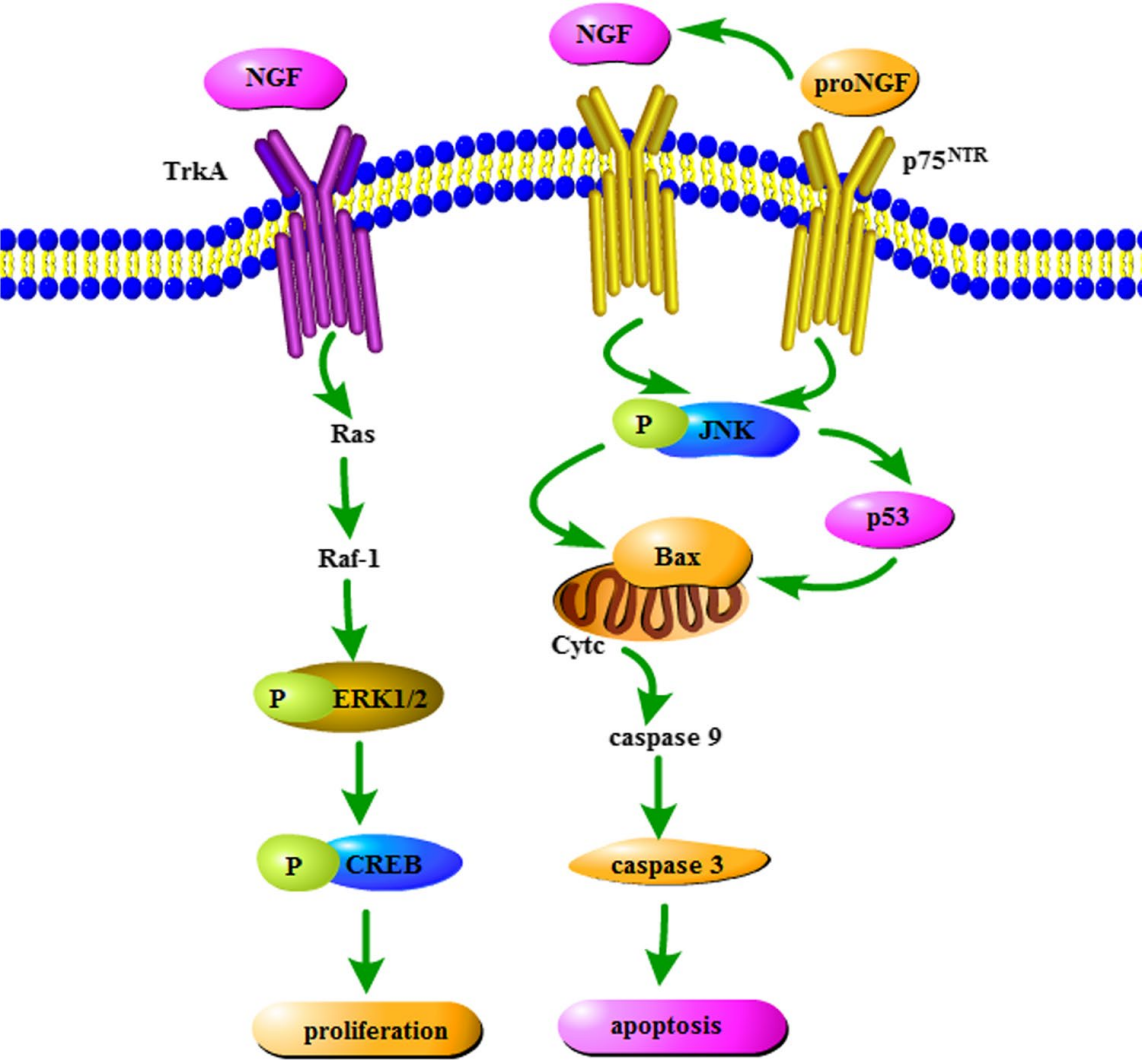
Effects of SCH and OHT on NGF-mediated TrkA and p75^NTR^ signaling pathway. NGF induces TrkA signaling pathway to increase neuronal proliferation. Pro-NGF, the precursor protein of NGF, can combine with p75^NTR^ preferentially to enhance neuronal apoptosis. CREB, cAMP response element-binding protein; JNK, C-Jun N-terminal kinase; NGF, nerve growth factor; OHT, overt hypothyroidism; p75^NTR^, p75 neurotrophin receptor; SCH, subclinical hypothyroidism; TrkA, tropomyosin-related kinase A

NGF can activate TrkA receptors and trigger three main signaling pathways: Ras/ERK, PLC-α, and PI3-kinase/Akt [41], then complete physiological activities, including neuronal survival, outgrowth and differentiation. ERK1/2 is the key to conducting signals from surface receptors to the nucleus. Active phosphorylation of ERK1/2 is involved in the proliferation, differentiation and apoptosis of neurons [42]. p-ERK1/2 can be translocated to the nucleus, where CREB is phosphorylated after interacting with other molecules [43, 44]. As an important molecule for amyloidosis, learning and memory, CREB plays a crucial role in neurodevelopment. Hence, we also detected the expression of p-CREB by immunofluorescence for further confirmation. In our experiments, TH deficiency decreased the amount of NGF, inhibited activity of TrkA, and decreased expression of p-ERK and p-CREB, which can be restored by timely supplementation with L-T_4_ to normal thyroid function during early pregnancy (Fig. 8).

The p75^NTR^ is a low-affinity receptor for NGF and binds with cell ligands to play a dual (proliferative/apoptotic) role in neurogenesis [45], and the JNK signaling pathway is a key step in this process [46]. p53, a genome guardian, is actuated by diverse stresses and stimuli and mediates DNA repair, apoptosis, and cell cycle arrest [47]. BAX, a key member of the Bcl-2 family of proteins, mediates apoptosis and releases cytochrome C from the mitochondrial membrane, through possible mechanisms that include selective disruption of the outer membrane as a result of mitochondrial matrix hyperpolarization/matrix swelling [48–50]. Apoptosomes are formed by cytochrome c with apoptotic protease activating factor-1(Apaf-1) and caspase-9 activates caspase-3 downstream [51]. As caspase-3 is a typical marker of apoptosis, we also detected its expression levels by immunofluorescence for further confirmation. In our experiments, TH deficiency increased the cleavage activity of p75^NTR^, activated JNK signaling, enhanced expression of p53, BAX and cleaved caspase-3, and increased neuronal apoptosis. These changes are reversed to normal thyroid function by timely supplementation with L-T_4_ in early pregnancy (Fig. 8).

## Conclusion

In conclusion, our study demonstrated that maternal SCH inhibits the TrkA signaling pathway, activated the p75^NTR^ signaling pathway, and unbalanced the TrkA/p75^NTR^ signaling pathway in the hippocampus of pups. This led to the decreased neuronal proliferation and increased neuronal apoptosis in offspring’s hippocampus, causing suboptimal neurocognitive ability. L-T_4_ treatment from early pregnancy could restore protein levels of the progeny to approximately normal levels, thereby ameliorating the adverse effects of maternal SCH on spatial learning and memory in offspring. We discovered that the offspring of subclinical hypothyroidism during pregnancy have abnormal neurodevelopment, and conducted preliminary research on the underlying mechanism and proposed some feasible directions for future research, such as how TSH and T_4_ affect the expression of the TrkA/p75^NTR^ signaling pathway.

## Data Availability

The datasets used and/or analyzed during the current study are available from the corresponding author on reasonable request. Data sharing not applicable to this article as no datasets were generated or analyzed during the current study.
